# Prevalence of depressive symptoms and knowledge, attitude, and practice among adolescents in Chengdu, China: a cross-sectional study

**DOI:** 10.3389/fpsyt.2025.1607695

**Published:** 2025-10-16

**Authors:** Xinze Jiang, Qinqin Zhao, Ruiying Zeng, Nisha Lei, Liping Wang

**Affiliations:** ^1^ Medical Affairs Department, Chengdu Shuangliu District Mental Health Center, Chengdu, Shuangliu, China; ^2^ Nursing Department, Chengdu Shuangliu District Mental Health Center, Chengdu, Shuangliu, China; ^3^ Infection Control Department, Chengdu Shuangliu District Mental Health Center, Chengdu, Shuangliu, China; ^4^ Moral Education Department, Chengdu Shuangliu District Education and Science Academy Affiliated Middle School, Chengdu, Shuangliu, China

**Keywords:** adolescents, depression, knowledge, attitudes, practice, mental health, self-rating depression scale, cross-sectional study

## Abstract

**Objective:**

Adolescent depression has emerged as a significant public health concern globally, including in China, and grasping a better understanding of adolescents’ views on depression could help design more adapted policies. This study aims to assess the prevalence of depressive symptoms among adolescents and examine their knowledge, attitudes, and practices (KAP) related to depression.

**Methods:**

A cross-sectional survey was conducted between September 23, 2024, and December 3, 2024, in primary and middle schools in Shuangliu District, Chengdu. Data were collected through self-administered questionnaires, which included demographic information, assessments of KAP regarding depression, and the Self-Rating Depression Scale (SDS). The possible attitude and practice scores ranged from 9 to 45, interpreted as negative (9-22), moderate (23-31), and positive (32-45).

**Results:**

A total of 541 valid questionnaires were analyzed. Of the respondents, 308 (56.93%) were female, and 109 (20.15%) were from single-parent households. SDS scores indicated that 18 participants (3.33%) exhibited depressive symptoms. The mean knowledge, attitude, practice, and SDS scores were 9.07±5.08 (possible range: 0-18), 33.37±4.28 (possible range: 9-45), 35.77±6.84 (possible range: 9-45), and 37.35±9.07, respectively. Mediation analysis showed that knowledge directly affected attitude (β = 0.128, P=0.019), attitude directly affected practice (β = 0.250, P=0.011), while SDS directly affected both attitude (β = -0.366, P=0.007) and practice (β = -0.637, P=0.008). Meanwhile, SDS has an indirect negative effect on practice (β = -0.090, P=0.005), and knowledge has an indirect positive effect on practice (β = 0.032, P=0.012).

**Conclusion:**

Adolescents in Chengdu had insufficient knowledge about depression while exhibiting positive attitudes and proactive practices, and most of them were without depressive symptoms. The study identified knowledge items that should be reinforced through educational interventions on adolescent depression. These findings emphasize the need for enhanced mental health education to improve adolescents’ understanding of depression, reinforce positive attitudes, and support proactive mental health practices.

## Introduction

Adolescent depression has emerged as a critical global public health concern, with systematic reviews indicating that 34% of adolescents worldwide experience elevated depressive symptoms, according to a meta-analysis published in 2022 (1). This prevalence has shown an alarming increase from 24% to 37% from 2000–2009 to 2010-2019 (2). The situation appears particularly concerning in Asian regions, where prevalence rates of depressive symptoms reach up to 40% (1). In China specifically, a recent large-scale epidemiological study found that major depressive disorder had a point prevalence of 8% and a lifetime prevalence of 19% among school-aged children and adolescents (3).

The adolescent period represents a crucial developmental stage marked by significant physiological, psychological, and social changes that can increase vulnerability to depression (4). A family history of depression significantly increases the risk for adolescents, with inherited genetic factors and intergenerational transmission mechanisms playing a major role (5). Exposure to psychosocial stress, including adverse childhood experiences, family conflict, bullying, and stigma (especially among LGBTQ2+ youth), increases vulnerability to depression (5). Adolescents face multiple social pressures, such as academic stress, peer relationships, and social isolation, which exacerbate depressive symptoms (6). Puberty and associated hormonal fluctuations, particularly increased estrogen in girls, heighten sensitivity to stress and contribute to the rise in depression rates during adolescence, especially among females. This hormonal influence partly explains why depression prevalence roughly doubles in girls compared to boys after age 13 (6). Structural factors such as lack of opportunities, poverty, ethnic minority status, and family socioeconomic challenges contribute to chronic stress and depression risk. For example, students in vocational schools, who often face more academic and employment pressures and poorer learning environments, show higher depression rates than those in general or key high schools (6). These factors increase the vulnerability of adolescents to depression; 50% of mental health disorders typically onset by age 14, and early-onset depression during adolescence presents with more severe manifestations in adulthood, characterized by longer episodes, higher recurrence rates, and more residual symptoms (1). These adverse outcomes extend to psychosocial domains, including lower educational attainment, unemployment, reduced perceived social support, and higher divorce rates (7, 8).

The knowledge-attitude-practice (KAP) theory plays a pivotal role in shaping human health behaviors (9). It is often employed alongside the KAP questionnaire to comprehensively gauge the knowledge, attitude, and practices of the target population within the healthcare domain, as well as to assess the demand and level of acceptance of relevant content (10). This model, integral to health literacy, is underpinned by the fundamental premise that knowledge exerts a positive influence on attitudes, and these attitudes, in turn, shape individual practices (11).

Despite the significant burden of adolescent depression, there are substantial gaps in mental health literacy among young people (12). Many adolescents struggle to correctly identify common mental health issues such as depression and anxiety. For example, in a study of adolescents in Delhi, only about 10% could accurately identify depression or anxiety from case vignettes (13). Similar findings have been reported in other contexts, including rural and urban youth in Malawi, where only 14% of those who knew someone with a mental illness could specify the disorder (14). High levels of stigma and misconceptions about mental health are pervasive. Nearly all adolescents in one study expressed stigmatizing attitudes toward depression and anxiety, which can discourage open discussion and support-seeking (13, 15). Understanding adolescents’ KAP regarding depression is crucial for developing targeted interventions, as misconceptions and stigma can create barriers to early identification and treatment (16). While some KAP studies have been conducted internationally, research specific to Chinese adolescents’ understanding and approaches to depression remains limited (17, 18). One study examined the parental KAP toward adolescent depression but not the status of the adolescents themselves (17), while the other investigated university students, who are young adults more than adolescents (18). The situation of adolescents in Chinese primary and secondary schools remain mostly unknown, and the present study aimed at addressing that gap.

Therefore, this study aims to assess the current state of adolescent depression and examine the KAP toward depression among Chinese adolescents. The findings will help identify knowledge gaps and misconceptions that could inform the development of more effective mental health education and intervention strategies tailored to this population’s specific needs and cultural context.

## Materials and methods

### Study design

This cross-sectional study was conducted between September 23, 2024, and December 3, 2024, in primary and middle schools in Shuangliu District, Chengdu.

### Participants

The study population consisted of adolescents who met the inclusion criteria. The inclusion criteria were (1) registered residency in Shuangliu District, Chengdu, and (2) age between 8 and 16 years. The 8–16 age range was selected in this study because, in China, children aged 8–16 years old are provided with access to mental health information as part of the standard social training curriculum. Therefore, they could understand the questionnaire. The selected schools were all part of a proactive psychological screening program conducted by our hospital. Students identified with psychological conditions during these screenings were already flagged for individualized intervention and were thus excluded from participation in the current study. Those individuals were excluded because their KAP would be “contaminated” by the interventions received for their psychological condition.

### Ethical considerations

Ethical approval was obtained from the Medical Ethics Committee of Gongxing Community Health Service Center, Shuangliu District, Chengdu (approval #20240601), and informed consent was secured from all participants prior to data collection. The consent process involved the research assistants, the school directors, the teachers, the parents, and the adolescents themselves. Before the questionnaire was distributed, the school notified the adolescents and their parents, and then the adolescents signed the informed consent form. The exact mechanical aspects of the consent process were left at the discretion of the school directors, but had to at least involve the people listed above.

### Sample size

The sample size was calculated using the formula for cross-sectional studies (19): α=0.05, $\text{n}={\left(\frac{Z_{1-\alpha /2}}{\delta }\right)}^{2}\times p\times (1-p)$ where $Z_{1-\alpha /2}$ =1.96 when α=0.05, an assumed degree of variability of p=0.5 maximizes the required sample size, and δ is admissible error (which was 5% here). The theoretical sample size was 461, which included an extra 20% to allow for problematic questionnaires that would have to be excluded.

### Questionnaire design

The questionnaire was developed by the investigators, and its development process involved multiple stages of validation. Initially, the instrument was designed based on a comprehensive literature review (20–22). The draft version underwent expert review by two specialists and was subsequently refined based on their feedback. A pilot study was conducted with 50 participants to assess the preliminary reliability, yielding a Cronbach’s α of 0.782. The final study included 541 questionnaires, with the overall Cronbach’s α coefficient improving to 0.809, demonstrating satisfactory internal consistency.

The final Chinese-language questionnaire comprised 60 items across five dimensions: basic information (13 items), depression status assessment (20 items), knowledge (9 items), attitude (9 items), and practice dimensions (9 items). The depression status was evaluated using the standardized Self-Rating Depression Scale (SDS), Chinese version, which shows an alpha coefficient of 0.89 (23). Responses were scored on a 4-point scale (1–4 or 4–1 points, depending on the item’s direction). The standard score was calculated using the formula: Standard Score = (Raw Score ÷ 80) × 100. Depression severity was categorized as follows: normal (<53), mild (53-62), moderate (63-72), and severe (≥73). For the knowledge dimension, responses were scored on a three-point scale: “Very familiar” (2 points), “Have heard of it” (1 point), and “Not clear” (0 points). The total possible scores ranged from 0 to 18. Knowledge level was categorized as insufficient (score <12), adequate (score 12–16), and excellent (score 16–18). Both attitude and practice dimensions employed 5-point Likert scales, with scoring adjusted according to item directionality. In the attitude dimension, items 1, 2, 5, 6, 8, and 9 were scored from 5 (very positive) to 1 (very negative), while items 3, 4, and 7 were reverse-scored. The total possible scores ranged from 9 to 45, with interpretations as follows: negative attitude (9-22), neutral attitude (23-31), and positive attitude (32-45). In the present study, practice refers to the actual behaviors or actions that adolescents take in relation to depressive symptoms and mental health. Similarly, the practice dimension used directional scoring: items 1–5 were scored from 1 to 5, while items 6–9 were reverse-scored. The total score ranged from 9 to 45, with classifications as negative practice (9-22), moderate practice (23-31), and positive practice (32-45).

### Questionnaire distribution and quality control

A team of five research assistants with medical backgrounds was recruited and trained for data collection. The questionnaire distribution was conducted in collaboration with three educational institutions in the Shuangliu District of Chengdu: the Education Institute Affiliated Middle School, the Education Institute Affiliated Primary School, and the Gongxing Middle School. The questionnaires were distributed to eligible students using convenience sampling in schools within Yixin Subdistrict, Shuangliu District. In each school, all students who met the inclusion criteria were provided with questionnaires for data collection. During the recruitment process, students were excluded if they did not provide signed informed consent or if they demonstrated an inability to understand the specific content of the questionnaire items. To ensure participant confidentiality, the questionnaires were designed to be anonymous, with personal identifiers removed, and informed consent forms collected separately from the survey responses. A total of >800 questionnaires were initially collected. After data cleaning, responses that were incomplete or contained responses to specific questions that were unclear or could not be properly interpreted were excluded, resulting in the final sample size used for analysis.

### Statistical methods

Data analysis was performed using SPSS 22.0 (IBM, Armonk, NY, USA). Continuous variables were presented as mean ± standard deviation (SD) if normally distributed, and comparisons between two groups were conducted using an independent-samples t-test. For non-normally distributed variables, data were expressed as the median (range), and comparisons were made using the Wilcoxon Mann-Whitney U test. When comparing three or more groups, one-way analysis of variance (ANOVA) was used for normally distributed variables with homogeneity of variance, while the Kruskal-Wallis test was applied for non-normally distributed variables. Categorical variables were expressed as frequencies and percentages (n, %). Pearson correlation analysis was performed to examine the relationships between depressive symptom status and KAP scores. The Pearson correlation coefficient ranged from -1 to +1, where positive values indicated a direct relationship, negative values suggested an inverse relationship, and a value of 0 implied no correlation. Structural equation modeling (SEM) was conducted to investigate the path relationships among depressive symptom status, knowledge, attitude, and practice. Model fit was assessed using standard goodness-of-fit indices, including the root mean square error of approximation (RMSEA), incremental fit index (IFI), Tucker-Lewis index (TLI), and comparative fit index (CFI). A two-sided P-value of less than 0.05 was considered statistically significant for all analyses.

## Results

### Demographic information on participants

**Figure 1:**
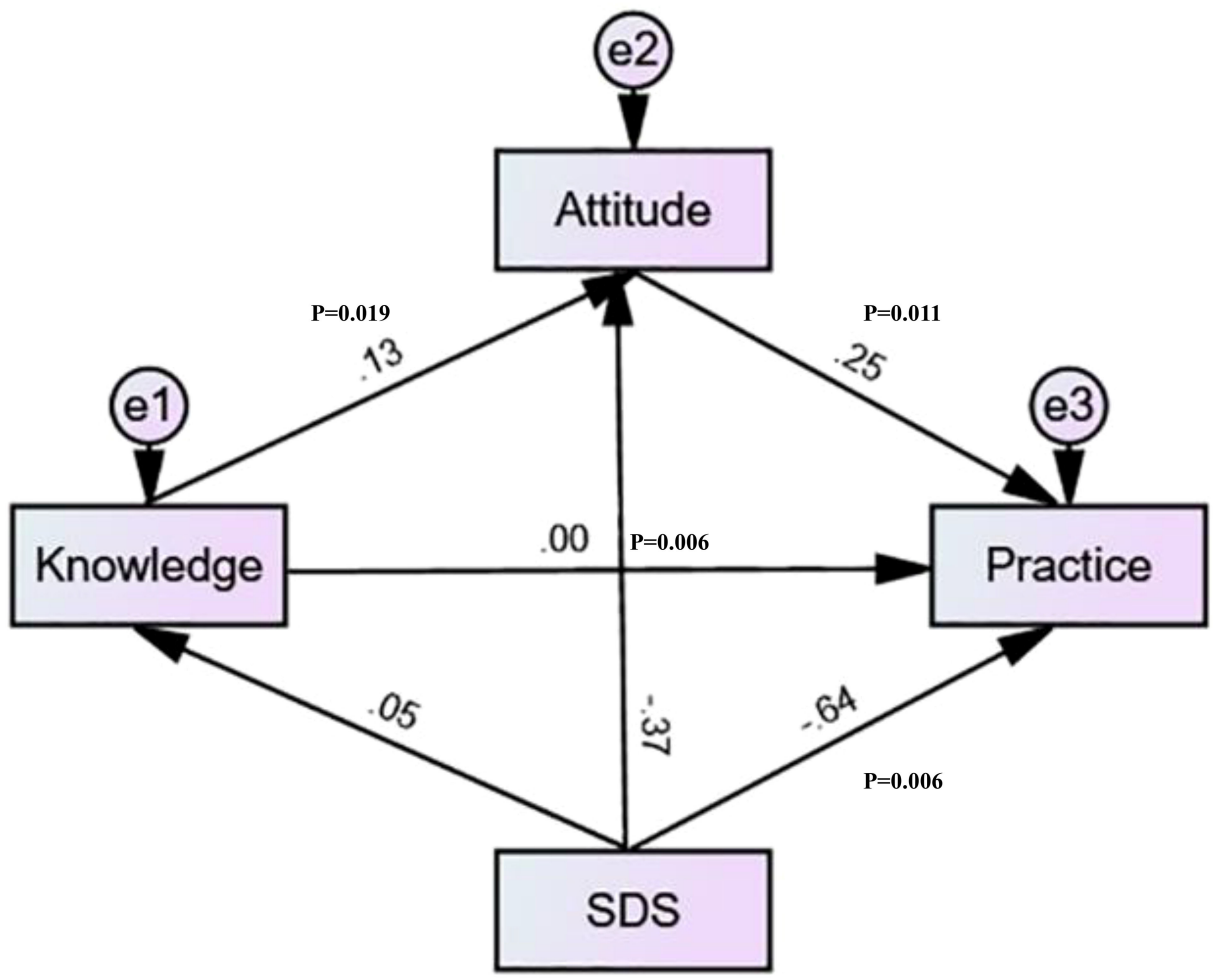
Participant flowchart.

**Figure 1** presents the participant flowchart. Out of 541 participants, 308 (56.93%) were female, 253 (46.77%) were the only child, 109 (20.15%) had a single-parent family, 318 (58.78%) had a very harmonious family atmosphere, and 388 (71.72%) had a good relationship with teachers and classmates. In addition, SDS scores showed that 18 (3.33%) had depressive symptoms.

### KAP and SDS scores

The mean knowledge, attitude, practice, and SDS scores were 9.07±5.08 (possible range: 0-18), 33.37±4.28 (possible range: 9-45), 35.77±6.84 (possible range: 9-45), and 37.35±9.07, respectively. The factors associated with the univariable analyses of the KAP and SDS scores are listed in **Table 1** and **Supplementary Table S1**.

**Table 1:** Demographic characteristics and KAP scores.

Variables	n (%)	Knowledge, mean ± SD	*P*	Attitude, mean ± SD	*P*	Practice, mean ± SD	*P*	SDS, mean ± SD	*P*
n=541		9.07 ± 5.08		33.37 ± 4.28		35.77 ± 6.84		37.35 ± 9.07	
School sources			0.135		**<0.001**		**<0.001**		**<0.001**
Affiliated Primary School of the Educational Science Institute	43 (7.95)	8.58 ± 5.86		36.09 ± 3.97		40.19 ± 5.39		34.23 ± 7.76	
Gongxing Middle School	146 (26.99)	9.85 ± 5.42		30.77 ± 3.79		32.59 ± 7.62		40.21 ± 9.87	
Affiliated Middle School of the Educational Science Institute	352 (65.06)	8.80 ± 4.81		34.11 ± 4.01		36.55 ± 6.10		36.55 ± 8.59	
Gender			**0.001**		0.074		**0.004**		0.124
Male	233 (43.07)	8.23 ± 5.33		33.70 ± 4.48		36.73 ± 6.48		36.55 ± 8.23	
Female	308 (56.93)	9.70 ± 4.80		33.11 ± 4.11		35.05 ± 7.02		37.95 ± 9.62	
Age			0.776		0.544		0.370		0.132
11-12	182 (33.64)	9.48 ± 5.40		33.31 ± 4.31		36.29 ± 7.80		36.36 ± 9.78	
13	184 (34.01)	8.68 ± 4.86		33.18 ± 4.22		35.24 ± 6.22		37.45 ± 9.03	
14	143 (26.43)	8.98 ± 5.00		33.81 ± 4.17		35.67 ± 6.35		38.61 ± 8.41	
15-16	32 (5.91)	9.31 ± 4.90		32.75 ± 4.84		36.28 ± 6.52		36.81 ± 7.42	
BMI			0.443		0.912		0.561		0.961
<18.5 Underweight	225 (41.59)	9.50 ± 5.64		33.46 ± 4.14		35.62 ± 7.15		37.50 ± 9.38	
18.5-24.9 Normal	259 (47.87)	8.81 ± 4.54		33.24 ± 4.45		35.68 ± 6.77		37.22 ± 8.81	
≥25 Overweight or Obesity	57 (10.54)	8.49 ± 5.07		33.56 ± 4.02		36.74 ± 5.83		37.37 ± 9.14	
Residence			0.262		**<0.001**		**<0.001**		**<0.001**
Rural	58 (10.72)	8.00 ± 4.33		31.16 ± 4.20		34.38 ± 7.61		35.86 ± 9.91	
Urban	227 (41.96)	8.96 ± 4.64		33.95 ± 4.22		37.63 ± 6.28		35.20 ± 8.15	
Suburban/Urban-rural fringe	256 (47.32)	9.40 ± 5.57		33.35 ± 4.19		34.44 ± 6.77		39.59 ± 9.15	
Ethnicity			0.971		0.151		0.430		0.869
Han	532 (98.34)	9.06 ± 5.06		33.33 ± 4.26		35.73 ± 6.85		37.35 ± 9.08	
Minority ethnic group	9 (1.66)	9.44 ± 6.64		35.22 ± 4.94		37.78 ± 6.00		37.44 ± 8.83	
The only child			0.158		0.061		0.191		0.663
Yes	253 (46.77)	9.44 ± 5.48		32.99 ± 4.24		35.31 ± 7.31		37.66 ± 9.75	
No	288 (53.23)	8.74 ± 4.69		33.70 ± 4.29		36.17 ± 6.38		37.08 ± 8.43	
Education			0.636		**<0.001**		**<0.001**		**<0.001**
Primary school	16 (2.96)	7.56 ± 5.40		28.81 ± 3.73		28.38 ± 3.79		45.38 ± 4.10	
Middle school	518 (95.75)	9.12 ± 5.10		33.55 ± 4.14		36.04 ± 6.77		37.03 ± 9.02	
High school/technical school	7 (1.29)	8.29 ± 1.70		30.14 ± 7.86		32.29 ± 7.54		42.86 ± 11.19	
Monthly income per capita, yuan (USD)*			**<0.001**		**0.035**		**<0.001**		**<0.001**
<2000 (<280)	32 (5.91)	14.63 ± 5.47		31.78 ± 3.13		26.94 ± 3.89		48.53 ± 4.96	
2000-5000 (280-700)	79 (14.6)	8.94 ± 4.57		34.08 ± 4.29		35.29 ± 6.04		38.41 ± 9.15	
5000-10,000 (700-1400)	124 (22.92)	8.30 ± 4.74		33.72 ± 4.34		36.98 ± 6.81		36.08 ± 9.78	
10,000-20,000 (1400-2800)	46 (8.5)	9.43 ± 5.18		33.96 ± 4.15		38.93 ± 5.95		34.07 ± 8.37	
>20,000 (>2800)	13 (2.4)	8.46 ± 6.31		33.38 ± 4.19		36.31 ± 4.96		34.69 ± 6.64	
Prefer not to disclose	247 (45.66)	8.74 ± 4.88		33.06 ± 4.35		35.84 ± 6.74		36.96 ± 8.25	
Family structure			0.548		**0.007**		**<0.001**		**<0.001**
Single-parent family	109 (20.15)	9.50 ± 6.43		32.16 ± 4.74		32.28 ± 7.33		42.29 ± 8.93	
Blended family	36 (6.65)	9.78 ± 4.50		32.92 ± 4.33		35.50 ± 6.83		38.47 ± 9.22	
Original two-parent family	389 (71.9)	8.86 ± 4.71		33.75 ± 4.06		36.77 ± 6.40		35.80 ± 8.52	
Other	7 (1.29)	10.43 ± 4.12		33.00 ± 5.10		36.00 ± 5.77		41.00 ± 12.45	
Smoking habit			**0.039**		0.292		**0.013**		**0.001**
Never smoked	532 (98.34)	8.99 ± 5.07		33.41 ± 4.25		35.88 ± 6.78		37.18 ± 9.03	
Used to smoke	3 (0.55)	11.33 ± 4.16		28.67 ± 6.51		26.33 ± 2.89		43.67 ± 4.04	
Currently smoking	6 (1.11)	14.33 ± 4.41		32.17 ± 5.00		30.33 ± 8.64		49.67 ± 0.82	
Drinking habit			0.585		0.261		0.109		**0.001**
Never drank	528 (97.6)	9.04 ± 5.11		33.43 ± 4.22		35.87 ± 6.80		37.17 ± 9.01	
Used to drink	6 (1.11)	9.33 ± 4.18		31.33 ± 5.96		32.83 ± 7.49		39.33 ± 10.37	
Currently drinking	7 (1.29)	10.86 ± 3.80		30.43 ± 5.97		30.86 ± 7.52		49.43 ± 1.51	
Family atmosphere			0.169		0.920		**<0.001**		**<0.001**
Very harmonious	318 (58.78)	9.39 ± 5.50		33.43 ± 4.44		36.86 ± 7.12		35.58 ± 9.18	
Relatively harmonious	199 (36.78)	8.60 ± 4.44		33.33 ± 4.12		34.66 ± 6.13		39.29 ± 8.11	
Frequent conflicts but reconcilable	20 (3.7)	9.30 ± 3.37		32.95 ± 3.39		30.15 ± 4.09		44.25 ± 8.53	
Tense and disharmonious, difficult to reconcile	4 (0.74)	5.25 ± 6.40		32.50 ± 2.52		31.75 ± 4.57		47.00 ± 8.04	
Relationship with teachers and classmates			**0.001**		0.825		**<0.001**		**<0.001**
Very good	388 (71.72)	9.62 ± 5.11		33.37 ± 4.36		36.52 ± 7.10		35.94 ± 9.21	
Average	139 (25.69)	7.65 ± 4.72		33.38 ± 4.15		34.05 ± 5.72		40.63 ± 7.73	
Somewhat tense	10 (1.85)	7.40 ± 5.17		33.50 ± 3.78		31.30 ± 4.52		41.80 ± 5.12	
Poor	4 (0.74)	9.25 ± 6.34		32.00 ± 1.41		33.75 ± 8.06		49.25 ± 4.43	
SDS			0.176		**0.008**		**<0.001**		–
<53 points (No depressive symptoms)	523 (96.67)	9.01 ± 5.12		33.46 ± 4.26		36.04 ± 6.70		–	
≥53 points (With depressive symptoms)	18 (3.33)	10.67 ± 3.73		30.78 ± 3.98		27.78 ± 5.95		–	

SD, standard deviation; BMI, body mass index; SDS, Self-Rating Depression Scale.

*The exchange rate is 1 CNY=0.14 USD.

The bold value indicates that the difference is statistically significant, P<0.05.

### Distribution of responses to the SDS standard self-rating scale, knowledge, attitude, and practice dimension

The distribution of the SDS standard self-rating scale showed that 54.34% were unable to feel as happy as usual when in close contact with the opposite sex (P6), 26.25% never or rarely felt the best in the morning of the day (P2), 22% reported not eating as much as they normally do (P5), and 19.04% never or rarely felt that making decisions was easy (P16) (**Supplementary Table S2**).

The distribution of knowledge dimensions showed that the three questions with the highest number of participants choosing the “Unclear” option were “If you notice significant and persistent abnormalities such as low mood in daily life, you can use the “9-Item Patient Health Questionnaire (PHQ-9)” for self-assessment” (K6) with 42.7%, “The causes of depression are unclear, but it is strongly associated with genetic, neurobiochemical, and psychosocial factors” (K5) with 33.27%, and “The goal of acute-phase treatment for depression is to control symptoms and achieve clinical remission (complete disappearance of symptoms) as much as possible” (K7) with 32.16% (**Supplementary Table S3**).

Responses to the attitude dimension showed that 15.16% strongly agreed and 9.06% agreed that schools lack educational courses or lectures on depression (A9), 8.5% strongly agreed, and 5.55% agreed that discussing their emotional problems is a sign of weakness (A4), and 7.02% strongly agreed, and 6.28% agreed that depression is simply “feeling bad” or “a lack of willpower” (A7) (**Supplementary Table S4**).

Responses to the practice dimension showed that 7.76% strongly agreed and 12.01% agreed that they have been more prone to losing their temper or becoming emotionally upset than usual (P2), 7.76% strongly agreed, and 9.61% agreed that their learning efficiency at school has significantly decreased (P1), and 7.02% strongly agreed and 3.14% agreed that they hide their depressive symptoms due to fear of discrimination (P5) (**Supplementary Table S5**).

### Correlation analysis

Further correlation analysis revealed very weak positive correlations between knowledge and attitude (r = 0.104, P=0.015) as well as between attitude and practice (r = 0.491, P < 0.001). Additionally, stronger negative correlations were found between attitude and SDS (r = -0.385, P < 0.001) as well as practice and SDS (r = -0.730, P < 0.001) (**Table 2**).

**Table 2:** Correlation analysis.

Dimensions	Knowledge	Attitude	Practice	SDS
Knowledge	1			
Attitude	0.104 (P=0.015)	1		
Practice	-0.005 (P=0.904)	0.491 (P<0.001)	1	
SDS	0.037 (P=0.384)	-0.385 (P<0.001)	-0.730 (P<0.001)	1

SDS, Self-Rating Depression Scale.

### Structural equation model

**Figure 2:**
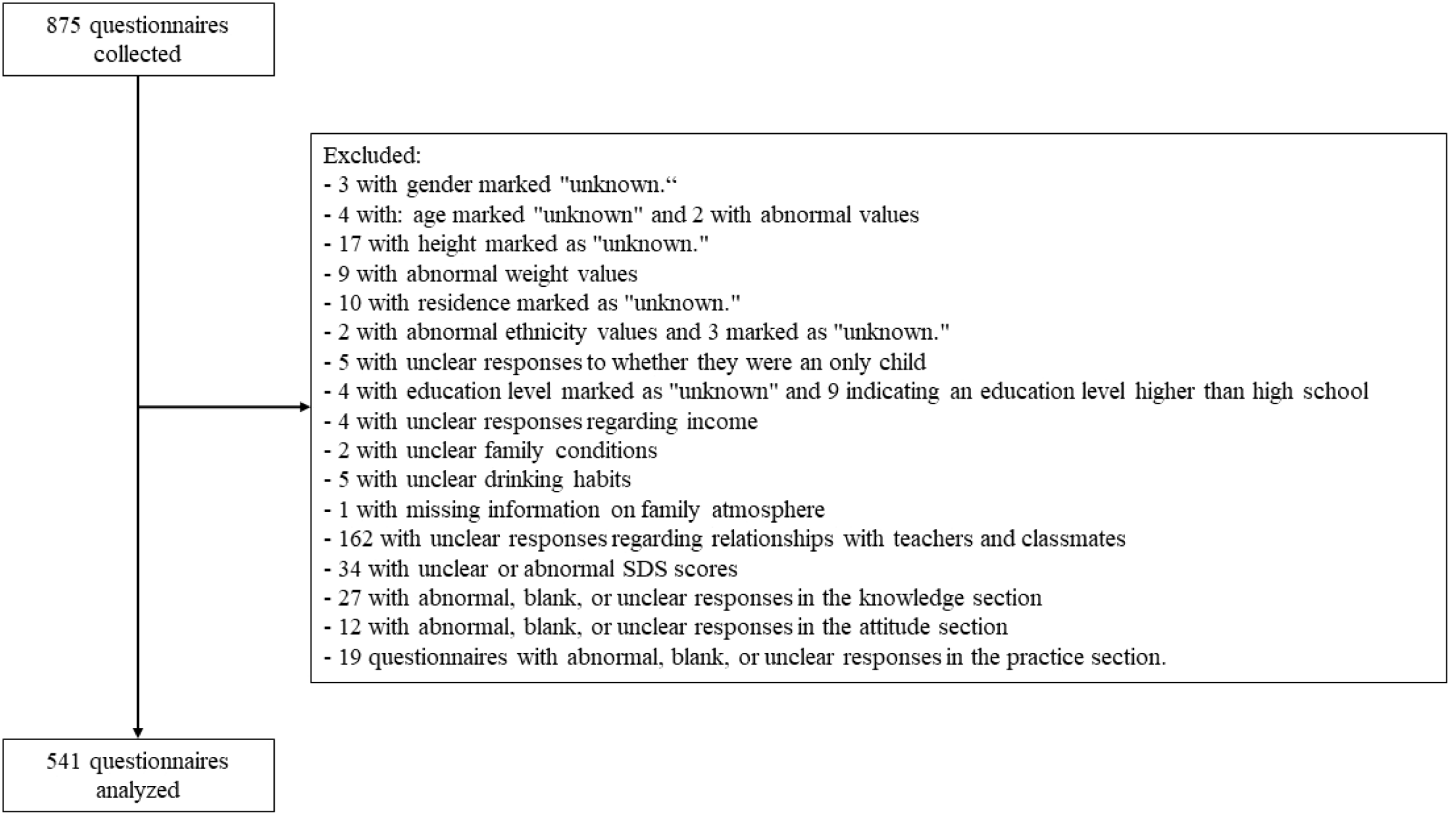
Structural equation modeling.

The structural equation model was established to investigate the effects of KAP and SDS further. The SDS scores negatively influenced the attitude and practice scores, while knowledge positively influenced attitude, and attitude positively influenced practice (**Table 3**). Mediation analysis showed that knowledge directly affected attitude (β = 0.128, P=0.019), attitude directly affected practice (β = 0.250, P=0.011), while SDS directly affected both attitude (β = -0.366, P=0.007) and practice (β = -0.637, P=0.008). Meanwhile, SDS has an indirect negative effect on practice (β = -0.090, P=0.005), and knowledge has an indirect positive effect on practice (β = 0.032, P=0.012) (**Table 4** and **Figure 2**).

**Table 3:** SEM total effect estimates.

Dimensions			Estimate	S.E.	C.R.	P
Knowledge	<—	SDS	0.028	0.024	1.169	0.242
Attitude	<—	Knowledge	0.108	0.033	3.210	0.001
Attitude	<—	SDS	-0.173	0.019	-9.202	<0.001
Practice	<—	Attitude	0.400	0.048	8.311	<0.001
Practice	<—	Knowledge	-0.002	0.038	-0.063	0.950
Practice	<—	SDS	-0.480	0.023	-21.292	<0.001

SDS, Self-Rating Depression Scale.

**Table 4:** Mediation analysis.

Model paths	Standardized total effects		Standardized direct effects		Standardized indirect effects	
	β (95%CI)	P	β (95%CI)	P	β (95%CI)	P
SDS→Knowledge	0.050 (-0.021~0.151)	0.130	0.050 (-0.021~0.151)	0.130		
SDS→Attitude	-0.360 (-0.442~-0.300)	0.006	-0.366 (-0.442~-0.303)	0.007	0.006 (-0.003~0.025)	0.110
SDS→Practice	-0.727 (-0.780~-0.678)	0.006	-0.637 (-0.693~-0.584)	0.008	-0.090 (-0.119~0.068)	0.005
Knowledge→Attitude	0.128 (0.043~0.197)	0.019	0.128 (0.043~0.197)	0.019		
Knowledge→Practice	0.030 (-0.025~0.085)	0.241	-0.002 (-0.056~0.197)	0.978	0.032 (0.010~0.053)	0.012
Attitude→Practice	0.250 (0.176~0.303)	0.011	0.250 (0.176~0.303)	0.011		

CI, confidence interval; SDS, Self-Rating Depression Scale.

## Discussion

Adolescents demonstrated insufficient knowledge about depression while exhibiting positive attitudes and proactive practices, and most of them were without depressive symptoms. These findings emphasize the need for strengthening depression-related education to further enhance adolescents’ knowledge, sustain positive attitudes, and promote effective mental health practices.

In the present study, the practice dimension toward depressive symptoms refers to the actual behavioral responses, coping mechanisms, and supportive actions undertaken by adolescents when experiencing or witnessing depressive symptoms. This dimension encompasses both potentially harmful behaviors (e.g., avoidance, concealment, loss of functioning) and positive mental health practices (e.g., help-seeking, peer support, advocacy, and awareness raising). The practice dimension assesses the behavioral responses and coping strategies of adolescents when faced with depressive symptoms in themselves or others. It captures both maladaptive and adaptive practices. The strong negative correlation between practice and SDS scores suggests that adolescents who actively engage in mental health-promoting behaviors tend to report fewer depressive symptoms. SEM further reinforced this relationship by demonstrating that depressive symptoms directly impaired mental health-related practices. Additionally, attitudes were shown to be a crucial mediating factor, with a strong positive association between attitude and practice, implying that adolescents with more favorable attitudes toward depression were more likely to adopt supportive mental health behaviors.

The prevalence of depression was 3.33% in the study population. The pooled prevalence of depressive symptoms among Chinese children and adolescents is estimated at approximately 22.2% to 26.2% (24). One recent meta-analysis that included 439 studies and nearly 1.5 million participants found a pooled point prevalence of 26.17% (95% CI: 25.00-27.41%) (25). Other meta-analyses and large studies have reported similar rates, with some regional or methodological variations (26). The low prevalence of depressive symptoms could be related to the fact that the participating schools were part of a psychological screening program conducted by the authors’ hospital. The students identified with psychological conditions were already flagged for individualized intervention and were thus excluded from participation in the study according to the exclusion criteria. Therefore, excluding the adolescents with identified mental health issues biased the prevalence results by underestimating the prevalence of depressive symptoms in the study population, limiting generalizability. Other reasons could also contribute to the low prevalence of depressive symptoms in the study population. Indeed, prevention services for depression among adolescents in Chengdu, China, integrate school-based interventions, specialized clinical facilities, community education programs, and public health initiatives focused on early detection and mental well-being. Chengdu has implemented positive education interventions in local schools. These programs aim to build resilience and prevent depression before symptoms arise, demonstrating effectiveness in preventing increases in depressive symptoms among adolescents (27). Many high schools, middle schools, and even university campuses in Chengdu (including the study institution) host mental health clinics. These clinics offer counseling, early screening, and psychoeducation to students. The Chengdu Mental Health Research Center for Youth, founded in 2018, provides comprehensive care for youth and families. Its services include psychological assessments, one-stop counseling, family education guidance, and support for socio-emotional development. It targets prevention as well as treatment and receives support from local health foundations and education associations. Each community district in Chengdu is served by a health service center, but only select centers provide dedicated mental health care for youth. These facilities may offer educational sessions, parental guidance, and referral pathways to specialized providers. Programs often target families to improve communication and education environments, recognizing the strong link between family dynamics and adolescent mental health. Workshops and resources for parents are part of community and clinical prevention strategies (28).

This pattern aligns with previous research indicating that proactive coping strategies and positive attitudes toward mental health issues contribute to lower depression risk among adolescents (29, 30). However, our results highlight a more nuanced dynamic, where knowledge alone does not directly influence practice but operates through attitude as a mediating factor. SEM results revealed that while knowledge significantly influenced attitude, its direct effect on practice was negligible, suggesting that simply increasing awareness of depression may not necessarily translate into behavioral changes unless it is accompanied by attitude shifts. This finding supports existing literature emphasizing that knowledge, though essential, must be paired with attitudinal interventions to foster meaningful behavioral engagement (31, 32).

Despite generally positive attitudes and active mental health practices, knowledge about depression remained limited, with rates of “very familiar” varying from 20.7% to 32.72%. Many adolescents lacked an understanding of specific symptoms, risk factors, and available treatment options, consistent with previous studies showing that while adolescents are often aware of mental health conditions in a broad sense, their knowledge of diagnostic criteria and intervention strategies is often superficial (33, 34). In contrast, attitudes were relatively favorable, with most students recognizing the seriousness of depression and acknowledging the importance of professional treatment. However, skepticism regarding treatment efficacy persisted among some students, reflecting findings from studies that mental health stigma has declined, but misconceptions about treatment remain prevalent (35, 36). A previous study of parents of adolescents in Ningbo City, China, regarding adolescent depression indicated moderate KAP scores, indicating that the knowledge they could share with their adolescents was limited.

The overall absence of significant depressive symptoms in the study population, as indicated by SDS scores, contrasts with findings from high-stress environments where adolescent depression rates are notably higher (37, 38). This may be attributed to contextual factors such as social support networks, educational pressures, and cultural perceptions of mental health. It is possible that while subclinical symptoms were present, adolescents in this study were not experiencing major depressive episodes. Nonetheless, the observed correlation between lower attitude and practice scores and higher SDS scores suggests that those with less favorable attitudes and fewer proactive behaviors may be at greater risk over time, reinforcing the need for early mental health education and intervention strategies. Of note, the adolescents who were already being followed for psychological issues were excluded, probably decreasing the observed prevalence. Nevertheless, this study suggests that the remaining proportion of undiagnosed psychological issues was low.

Socioeconomic disparities also played a role in shaping KAP dimensions and SDS scores. Adolescents from lower-income households demonstrated higher knowledge scores but lower practice scores and higher SDS scores, suggesting that economic disadvantage may heighten awareness of mental health challenges while simultaneously limiting access to resources necessary for effective coping. This pattern has been observed in other studies where financial constraints create a barrier to enacting positive health behaviors (39, 40). Additionally, differences across schools indicated that variations in educational programs, school environments, and access to mental health resources may contribute to differences in KAP and SDS scores. Similar findings have been reported in studies showing that school climate and access to counseling services significantly impact adolescent mental health outcomes (41, 42).

Findings from the distribution analyses further contextualized these trends. Responses from the SDS self-rating scale revealed that while severe depressive symptoms were rare, many students reported occasional negative emotions, such as low energy, difficulty concentrating, and mild feelings of sadness. These findings are consistent with research suggesting that while full-scale clinical depression is uncommon in general adolescent populations, subclinical symptoms are widespread and may still impact academic performance and social functioning (43, 44).

In terms of knowledge, while a majority of students had heard of core depression symptoms, many lacked familiarity with diagnostic tools and available treatments. This is consistent with previous research showing that adolescents, despite increasing exposure to mental health topics, often remain unaware of specific self-assessment methods or professional treatment options (45, 46). Attitudinal responses reflected a growing acceptance of mental health discussions, but a significant proportion of students remained neutral or skeptical about treatment efficacy. This highlights a gap between recognition of mental health conditions and willingness to seek or advocate for treatment.

Addressing these gaps requires a multifaceted approach. Educational interventions should focus not only on increasing knowledge but also on strengthening positive attitudes, as attitude plays a critical mediating role in translating knowledge into practice. Interactive learning experiences, such as role-playing, storytelling, and peer-led discussions, could be incorporated into school curricula to encourage engagement and reinforce positive attitudes. Schools should also integrate mental health discussions into broader wellness education rather than presenting them as isolated topics, as this may normalize conversations around mental health and encourage more students to seek help when needed (47, 48).

Additionally, targeted interventions for students from lower-income households should be considered, such as providing free or subsidized mental health services in schools and communities. Given the disparities observed across different schools, school-based interventions should be tailored to specific institutional needs, with schools that exhibit lower practice scores and higher SDS scores receiving prioritized access to counseling services and mental health education programs. Expanding access to trained school counselors, integrating mental health education into national curricula, and implementing policies that encourage early screening and intervention could significantly enhance outcomes (49, 50).

China has implemented a comprehensive and multi-layered set of interventions to improve the mental well-being of adolescents, reflecting a growing recognition of the importance of youth mental health at both policy and practice levels. Among the national policy initiatives, the Special Action Plan for Comprehensively Strengthening and Improving the Mental Health Work of Students in the New Era (2023–2025) and the Healthy China Action Plan (2019–2030) include specific mandates for schools to establish psychological service platforms, improve access to mental health services, and promote awareness and early intervention among children and adolescents (51). School-based and community interventions are also performed, including school mental health services, peer support programs, and standardized mental health curricula (51–53). There is a growing emphasis on connecting schools with mental health professionals and hospitals, which are being guided to establish psychological counseling clinics for children and adolescents, increasing access to professional care. Programs like the WHO’s Step-by-Step (SbS) digital intervention have been adapted for Chinese youth, delivering evidence-based psychological support via apps and online platforms. These tools provide guided self-help, mood regulation strategies, and remote peer support, making mental health care more accessible, especially in areas with workforce shortages (54). New national systems are being developed to monitor student mental health and provide early warnings, leveraging digital technologies for broad, scalable impact (51). National and provincial experts conduct lectures and outreach activities in schools and communities to raise awareness, reduce stigma, and promote positive mental health practices. The government is investing in training more school counselors and mental health professionals and allocating special funds to support prevention, early intervention, and treatment programs. The present study provides KAP data from the adolescents’ perspective. Such data should be considered when revising the policies to make sure they align with the needs of the adolescents.

A β-value of 0.13 between knowledge and attitude indicates a positive but weak association between knowledge and attitude, suggesting that as knowledge increases, attitudes tend to improve slightly, which can then lead to more positive practices. However, the relationship between knowledge and attitude is not absolute, and attitudes can, in fact, be influenced or changed without a corresponding increase in knowledge. Indeed, research and practical experience in behavior change show that while knowledge is often the first step, it is not always sufficient to change practice on its own (55–57). Attitudes are influenced by a variety of factors, including personal experiences, emotions, social influences, and the perceived relevance of information. For example, interventions that focus on emotional engagement, direct experience, social norms, or motivational factors can shift attitudes even if knowledge remains unchanged (55–57). Moreover, attitudes can be formed or altered through mechanisms such as direct personal experience with the subject, exposure to persuasive communications or social influence, changes in the social or cultural environment, and emotional or motivational appeals that do not necessarily impart new knowledge but reshape how people feel about an issue (55–57).

The strengths of the study included the fact that public health is important. The method of data analysis used in this manuscript is helpful in uncovering how and why one variable affects another, revealing the underlying process (mediation analysis). It enhances the planning of intervention strategies. In addition, the instruments were locally adapted.

This study has several limitations. First, the cross-sectional design prevents the establishment of causal relationships between knowledge, attitudes, practices, and depressive symptoms. Second, the study was performed in a single city in China, limiting generalizability. There are important differences in adolescent depression prevalence and risk factors between China and other countries, highlighting the need to perform similar studies elsewhere for comparison and to improve the knowledge pertaining to adolescent depression. The factors that can influence adolescent depression in countries include socioeconomic status, cultural factors, urbanization, gender differences, parenting styles, and stigma and awareness (58–60). Third, the use of self-reported questionnaires may introduce response bias, recall bias, and social desirability bias, as participants might underreport or overreport their symptoms and perceptions, influenced by social expectations. Fourth, the study was conducted in a single district, which may limit the generalizability of the findings to broader adolescent populations in China or in other countries or areas. Fifth, adolescents with severe depression could have been absent from school when the questionnaire was distributed, leading to some bias. Furthermore, as stated in the Methods, adolescents already identified as having psychological issues were excluded. Finally, SEM fit indices could not be provided. Indeed, the model was a saturated model, in which the number of parameters to be estimated equals the number of unique data points (variance-covariance elements) provided by the observed data. As a result, the model has degrees of freedom (df) = 0, meaning that it is just identified and perfectly fits the observed data by design. In a saturated model, because df = 0, the chi-squared value also equals 0 (since chi-square = (fit function value) × df), and other fit indices that are dependent on the chi-squared value or df, such as RMSEA, CFI, and TLI, become either 0 or 1, losing their interpretative value. Specifically, in our case, this resulted in RMSEA = 0 (indicating a perfect fit, though meaningless in this context), CFI = 1, and Tucker-Lewis index = 1. Thus, model fit indices are not informative or applicable in the case of a saturated model.

Furthermore, several confounding factors can influence adolescent depression, including biological and genetic factors (e.g., family history of depression, sex and puberty, and physical illness or disability), socioeconomic and demographic factors (e.g., socioeconomic status and migrant background and minority status), psychological and cognitive factors (e.g., cognitive vulnerabilities and co-occurring anxiety symptoms), social and environmental factors (e.g., family environment, peer relationships and bullying, and social support), life events and stress (e.g., acute and chronic stressors and substance use), and lifestyle and health-related factors (e.g., physical activity and sleep and chronic diseases) (5, 61–63). Several of those confounders were addressed in the present study, but some could not be determined because they could be unreliable when self-reported, subject to privacy issues, or would entail additional questionnaires that would decrease the response rate.

## Conclusion

The SDS scores indicated that 3.33% of the participants exhibited depressive symptoms. Mediation analysis showed that knowledge directly affected attitude, attitude directly affected practice, while SDS directly affected both attitude and practice. Meanwhile, SDS has an indirect negative effect on practice, and knowledge has an indirect positive effect on practice. Therefore, adolescents demonstrated insufficient knowledge about depression but exhibited generally positive attitudes and proactive practices. The overall SDS scores suggest an absence of significant undiagnosed depressive symptoms in the surveyed population since individuals with diagnosed psychological issues were excluded. These findings highlight the need to strengthen depression-related education among adolescents to enhance their knowledge, maintain positive attitudes, and promote effective mental health practices, thereby supporting long-term psychological well-being. Knowledge influences practice through attitude, which should be considered when designing interventions in the future. Future research should evaluate the effectiveness of KAP-based interventions.

## Data Availability

The original contributions presented in the study are included in the article/Supplementary Material. Further inquiries can be directed to the corresponding author.
