# Supplementary material for: Prevalence of depressive symptoms and knowledge, attitude, and practice among adolescents in Chengdu, China: a cross-sectional study
Source: Front Psychiatry. 2025 Oct 16;16:1607695. doi: 10.3389/fpsyt.2025.1607695 (PMC12575669; doi:10.3389/fpsyt.2025.1607695)
Supplement: Supplementary file 1 [file DataSheet1.docx]

**Supplementary Table S1. Matrix layout of the significant associations of variables with KAP and SDS scores.**

|  | **Knowledge** | **Attitude** | **Practice** | **SDS** |
| --- | --- | --- | --- | --- |
| **School sources** | \ | *** | *** | *** |
| **Gender** | ** | \ | ** | \ |
| **Residence** | \ | *** | *** | *** |
| **Education** | \ | *** | *** | *** |
| **Monthly income per capita** | *** | * | *** | *** |
| **Family structure** | \ | ** | *** | *** |
| **Smoking habit** | * | \ | * | \ |
| **Drinking habit** | \ | \ | \ | ** |
| **Family atmosphere** | \ | \ | *** | *** |
| **Relationship with teachers and classmates** | ** | \ | *** | *** |
| **SDS** | \ | ** | *** | \ |

* p<0.05, ** p<0.01, ***p<0.001

**Supplementary Table S2. SDS standard self-rating scale**

|  | **n (%)** | | | |
| --- | --- | --- | --- | --- |
|  | **None or very little of the time** | **Some of the time** | **A good part of the time** | **Most or all of the time** |
| **1.** **I feel downhearted, blue, and in low spirits** | 344 (63.59) | 172 (31.79) | 23 (4.25) | 2 (0.37) |
| **2.** **I feel best in the morning** | 142 (26.25) | 179 (33.09) | 125 (23.11) | 95 (17.56) |
| **3.** **I sometimes feel like crying or feel the urge to cry** | 392 (72.46) | 117 (21.63) | 24 (4.44) | 8 (1.48) |
| **4.** **I have trouble sleeping at night** | 343 (63.4) | 146 (26.99) | 33 (6.1) | 19 (3.51) |
| **5.** **I eat as much as I normally do** | 119 (22) | 62 (11.46) | 118 (21.81) | 242 (44.73) |
| **6.** **I feel as happy as usual when in close contact with the opposite sex** | 294 (54.34) | 121 (22.37) | 67 (12.38) | 59 (10.91) |
| **7.** **I find that I am losing weight** | 428 (79.11) | 93 (17.19) | 14 (2.59) | 6 (1.11) |
| **8.** **I am bothered by constipation** | 461 (85.21) | 55 (10.17) | 18 (3.33) | 7 (1.29) |
| **9. My heart beats faster than usual** | 376 (69.5) | 143 (26.43) | 18 (3.33) | 4 (0.74) |
| **10. I feel fatigued for no reason** | 328 (60.63) | 145 (26.8) | 48 (8.87) | 20 (3.7) |
| **11. My mind is as clear as usual** | 85 (15.71) | 69 (12.75) | 143 (26.43) | 244 (45.1) |
| **12. I find that it’s not difficult to do things I often do** | 96 (17.74) | 139 (25.69) | 173 (31.98) | 133 (24.58) |
| **13. I feel restless and cannot calm down** | 329 (60.81) | 158 (29.21) | 40 (7.39) | 14 (2.59) |
| **14. I am hopeful about the future** | 74 (13.68) | 85 (15.71) | 149 (27.54) | 233 (43.07) |
| **15. I get easily upset or irritated** | 339 (62.66) | 139 (25.69) | 44 (8.13) | 19 (3.51) |
| **16.I find it easy to make decisions** | 103 (19.04) | 181 (33.46) | 174 (32.16) | 83 (15.34) |
| **17. I feel like I am a useful person and needed by others** | 83 (15.34) | 145 (26.8) | 166 (30.68) | 147 (27.17) |
| **18. My life is meaningful** | 70 (12.94) | 98 (18.11) | 160 (29.57) | 213 (39.37) |
| **19. I think others would live better if I were dead** | 430 (79.48) | 67 (12.38) | 27 (4.99) | 17 (3.14) |
| **20. I am as interested as usual in things I normally enjoy** | 96 (17.74) | 75 (13.86) | 157 (29.02) | 213 (39.37) |

**Supplementary Table S3. Distribution of knowledge dimension responses**

|  | **n (%)** | | |
| --- | --- | --- | --- |
|  | **Very familiar** | **Heard of it** | **Unclear** |
| **1.The core symptoms of depression are significant and persistent low mood and loss of interest** | 177 (32.72) | 265 (48.98) | 99 (18.3) |
| **2.Symptoms of a depressive episode include low mood, slowed thinking, and decreased volitional activity** | 177 (32.72) | 256 (47.32) | 108 (19.96) |
| **3.Depressed individuals lose interest or enthusiasm for everything, lose passion for their previous hobbies, and are unable to experience joy** | 176 (32.53) | 251 (46.4) | 114 (21.07) |
| **4.Symptoms of depression are varied, and there are individual differences in how each patient presents** | 149 (27.54) | 258 (47.69) | 134 (24.77) |
| **5.The causes of depression are unclear, but it is strongly associated with genetic, neurobiochemical, and psychosocial factors** | 138 (25.51) | 223 (41.22) | 180 (33.27) |
| **6.If you notice significant and persistent abnormalities such as low mood in daily life, you can use the "9-Item Patient Health Questionnaire (PHQ-9)" for self-assessment** | 112 (20.7) | 198 (36.6) | 231 (42.7) |
| **7.The goal of acute-phase treatment for depression is to control symptoms and achieve clinical remission (complete disappearance of symptoms) as much as possible** | 122 (22.55) | 245 (45.29) | 174 (32.16) |
| **8.Psychological treatment for depression mainly involves talking and communicating with doctors. Depending on the issues addressed, it can include supportive psychotherapy, cognitive behavioral therapy, psychodynamic therapy, interpersonal therapy, and marital or family therapy** | 157 (29.02) | 258 (47.69) | 126 (23.29) |
| **9. Antidepressant medication is effective for treating depressive mood, as well as accompanying anxiety, tension, and physical symptoms** | 142 (26.25) | 251 (46.4) | 148 (27.36) |

**Supplementary Table S4. Distribution of attitude dimension responses**

|  | **n (%)** | | | | |
| --- | --- | --- | --- | --- | --- |
|  | **Strongly agree** | **Agree** | **Neutral** | **Disagree** | **Strongly disagree** |
| **1.You think depression is a serious mental illness** | 201 (37.15) | 127 (23.48) | 177 (32.72) | 20 (3.7) | 16 (2.96) |
| **2.You think depression patients can recover through treatment** | 143 (26.43) | 160 (29.57) | 202 (37.34) | 24 (4.44) | 12 (2.22) |
| **3.You often suspect you might have depression** | 37 (6.84) | 27 (4.99) | 125 (23.11) | 115 (21.26) | 237 (43.81) |
| **4.You think discussing your emotional problems is a sign of weakness** | 46 (8.5) | 30 (5.55) | 177 (32.72) | 119 (22) | 169 (31.24) |
| **5.You think depression patients need professional treatment** | 252 (46.58) | 142 (26.25) | 128 (23.66) | 10 (1.85) | 9 (1.66) |
| **6.You strongly dislike the prejudice or discrimination others show toward depression** | 265 (48.98) | 105 (19.41) | 114 (21.07) | 21 (3.88) | 36 (6.65) |
| **7.You think depression is simply "feeling bad" or "a lack of willpower”** | 38 (7.02) | 34 (6.28) | 183 (33.83) | 150 (27.73) | 136 (25.14) |
| **8.You think depression is associated with suicide risk** | 124 (22.92) | 177 (32.72) | 201 (37.15) | 19 (3.51) | 20 (3.7) |
| **9.You think schools lack educational courses or lectures on depression** | 82 (15.16) | 49 (9.06) | 212 (39.19) | 91 (16.82) | 107 (19.78) |

**Supplementary Table S5. Distribution of practice dimension responses**

|  | **n (%)** | | | | |
| --- | --- | --- | --- | --- | --- |
|  | **Strongly agree** | **Agree** | **Neutral** | **Disagree** | **Strongly disagree** |
| **1.Over the past month, you’re learning efficiency at school has significantly decreased** | 42 (7.76) | 52 (9.61) | 145 (26.8) | 141 (26.06) | 161 (29.76) |
| **2.Over the past month, you have been more prone to losing your temper or becoming emotionally upset than usual** | 42 (7.76) | 65 (12.01) | 133 (24.58) | 121 (22.37) | 180 (33.27) |
| **3.Over the past month, you have often avoided interactions with friends, family, or classmates** | 31 (5.73) | 25 (4.62) | 116 (21.44) | 133 (24.58) | 236 (43.62) |
| **4.When you feel low, the mood usually lasts for a long time** | 33 (6.1) | 43 (7.95) | 145 (26.8) | 111 (20.52) | 209 (38.63) |
| **5.You hide your depressive symptoms due to fear of discrimination** | 38 (7.02) | 17 (3.14) | 128 (23.66) | 105 (19.41) | 253 (46.77) |
| **6.When you encounter difficulties or feel upset, you confide in or seek help from others (e.g., family, friends, teachers)** | 214 (39.56) | 127 (23.48) | 145 (26.8) | 39 (7.21) | 16 (2.96) |
| **7.If someone around you is experiencing depression, you encourage them to seek treatment** | 364 (67.28) | 88 (16.27) | 79 (14.6) | 4 (0.74) | 6 (1.11) |
| **8.You make an effort to challenge the prejudice against adolescent depression in your surroundings** | 295 (54.53) | 97 (17.93) | 140 (25.88) | 2 (0.37) | 7 (1.29) |
| **9.You actively work to raise awareness among those around you, including parents, about the importance of adolescent depression issues** | 294 (54.34) | 108 (19.96) | 125 (23.11) | 6 (1.11) | 8 (1.48) |
